# Patterns of HIV and SARS‐CoV‐2 co‐infection in Wuhan, China

**DOI:** 10.1002/jia2.25568

**Published:** 2020-07-01

**Authors:** Wei Guo, Fangzhao Ming, Yong Feng, Qian Zhang, Pingzhen Mo, Lian Liu, Ming Gao, Weiming Tang, Ke Liang

**Affiliations:** ^1^ Department of Pathology Zhongnan Hospital of Wuhan University Wuhan China; ^2^ Department of Pathology School of Basic Medical Sciences Wuhan University Wuhan China; ^3^ Wuchang District Center for Disease Control and Prevention Wuhan China; ^4^ State Key Laboratory of Virology/Department of Medical Microbiology School of Basic Medical Sciences Wuhan University Wuhan China; ^5^ Qingshan District Center for Disease Control and Prevention Wuhan China; ^6^ Department of Infectious Diseases Zhongnan Hospital of Wuhan University Wuhan University Hubei China; ^7^ Caidian District Center for Disease Control and Prevention Wuhan China; ^8^ Xinzhou District Center for Disease Control and Prevention Wuhan China; ^9^ Dermatology Hospital Southern Medical University, and the University of North Carolina at Chapel Hill Project‐China Guangzhou China

**Keywords:** clinical patterns, HIV, SARS‐CoV‐2, Co‐infection, COVID‐19, CD4^+^ T lymphocyte count, lymphopenia

People living with HIV/AIDS (PLWHA) were initially considered to be at an increased risk of infection with SARS‐CoV‐2 due to their risk of immunosuppresion [1]. However, few studies have reported on co‐infections between HIV and SARS‐CoV‐2 and treatment outcomes, and data to date do not support either increased acquisition or severity of COVID‐19 [2, 3]. Here we present the clinical and laboratory patterns in 14 co‐infected cases, including three asymptomatic SARS‐CoV‐2 carriers in Wuhan, China, from 16 February to 30 April 2020.

The 14 cases were coded P1 to P14 in order of their infection severity, namely: critical (P1 and P2), severe (P3 to P5), mild (P6 to P11) and asymptomatic (P12 to P14) (Table 1). The case group consisted of 13 men who have sex with men (MSM) and one heterosexual female. Only P10 and P13 had smoking history, and none of them abused alcohol.

**Table 1:** Clinical features of the COVID‐19 patients living with HIV in Wuhan, China, 2020 (N = 14)

	Patient 1^a^	Patient 2^a^	Patient 3	Patient 4	Patient5	Patient 6	Patient7	Patient8	Patient 9	Patient 10	Patient 11	Patient 12^b^	Patient 13^b^	Patient 14^b^
**Demographic and baseline features of the patients**														
Ages (years)	56	68	60	58	38	31	71	63	44	51	44	24	57	64
Sex	Male	Male	Male	Female	Male	Male	Male	Male	Male	Male	Male	Male	Male	Male
HIV‐risk factor	MSM	MSM	MSM	Heterosexual	MSM	MSM	MSM	MSM	MSM	MSM	MSM	MSM	MSM	MSM
Comorbidities	COPD	Hypertension	Diabetes Lymphoma	Hypertension Atrial fibrillation	PCP	None	Hypertension	HypertensionCerebral infarction	None	Hypertension	None	Kaposi's Sarcoma	Bronchiectasia Pulmonary tuberculosis Anemia	None
ART regimen	AZT + 3TC+EFV	AZT + 3TC+NVP	TDF + 3TC+EFV	RPV + TDF+FTC	None	AZT + 3TC+NVP	AZT + 3TC+NVP	AZT + 3TC+EFV	EFV + 3TC+TDF	AZT + 3TC+NVP	Lpv/r + 3TC+TDF	EVG/c + FTC+TAF	AZT + 3TC+EFV	TDF + 3TC+EFV
Latest CD4 count (cells/µL)	695	350	141	722	34	420	586	169	506	817	198	29	750	302
Latest HIV‐VL (copies/mL)	<20	<20	<20	<20	35600	<20	<20	<20	<20	<20	<20	–	<20	452
**Clinical features on admission**														
Diagnosis symptoms														–
Fever	+	+	+	+	+	+	–	+	+	+	+	–	–	–
Cough	+ (dry)	+(dry)	+ (dry)	+ (dry)	–	+ (dry)	+ (dry)	–	–	+ (dry)	–	–	–	–
Dyspnoea	+	+	+	+	+	+	–	–	–	–	+	–	–	–
Fatigue	+	+	+	+	+	+	–	+	+	+	+	–	+	–
Blood pressure (mmHg)	98/45	133/90	117/69	139/79	145/93	139/87	109/95	153/97	130/80	135/80	116/84	124/80	102/75	130/86
Body temperature (°C)	38.6	38.5	39.3	38.9	39.5	40.0	36.8	38.7	38.6	37.5	39.8	Normal	Normal	Normal
Oximetry saturation	76%	82%	90%	87%	91%	94%	96%	97%	96%	98%	90%	99%	98%	100%
Chest CT scan	GGO	GGO	GGO	Bilateral patchy shadowing	GGO	Bilateral patchy shadowing	GGO	GGO	GGO	GGO	GGO	Normal	Bronchiectasia Bilateral pleural effusion	Normal
WBC (×10^9^ cells/L) (normal range 3.5 to 9.5)	4.36	6.7	3.41	2.29	4.2	1.83	4.51	1.65	3.1	4.44	5.2	4.32	2.16	7.04
LYM (×10^9^ cells/L) (normal range 1.1 to 3.2)	1.1	0.35	0.9	0.62	1.55	0.95	1.45	0.41	1.6	1.86	0.68	1.22	1.13	1.44
PLT (×10^9^ cells/L) (normal range 125 to 350)	360	65	95	132	267	120	168	118	234	165	232	383	111	210
IL‐6 (pg/mL) (normal range 0 to 7)	–	18.62	–	–	9.87	14.08	–	–	–	10.21	–	–	3.25	–
Clinical classification	Critical	Critical	Severe	Severe	Severe	Mild	Mild	Mild	Mild	Mild	Mild	Asymptomatic carrier	Asymptomatic carrier	Asymptomatic carrier
**Treatment and clinical outcomes**														
Admitted to ICU	None	Yes	None	None	None	None	None	None	None	None	None	None	None	None
Mechanical ventilation	None	Yes	None	None	None	None	None	None	None	None	None	None	None	None
Anti‐virus Therapy	None	None	Oseltamivir	Abidol	Abidol	Oseltamivir	None	None	None	None	None	None	None	Abidol
Antibiotic Therapy	Yes	Yes	Yes	Yes	Yes	Yes	Yes	No	Yes	Yes	Yes	None	None	None
Corticosteroids	None	Yes	None	Yes	Yes	None	None	None	None	None	None	None	None	None
Prognosis	Death	Death	Cure	Cure	Cure	Cure	Cure	Cure	Cure	Cure	Cure	Cure	Cure	Cure
Length of hospital stay (days)	1	6	18	21	47	11	8	9	24	14	23	10	14	7
Duration of positive nucleic acid test (days)	–	–	12	17	10	6	6	8	21	10	15	7	4	5

3TC, Lamivudine; ART, antiretroviral treatment; AZT, Zidovudine; COPD, chronic obstructive pulmonary disease; EFV, Efavirenz; EVG/c, Elvitegravir/cobi; FTC, Emtricitabine; GGO, ground glass opacity; HIV‐VL, HIV viral load; ICU, intensive care unit.; LPV/r, Lopinavir/Ritonavir; LYM, lymphocytes; MSM, men who have sex with men; NVP, Nevirapine; PCP, pneumocystis pneumonia; PLT, platelet; RPV, Rilpivirine; TDF, Tenofovir; WBC, white blood cells.

^a^ The two patients died

^b^ Asymptomatic carrier.

The median age of the COVID‐19 patients was 56 (range 31 to 71 years old), whereas the three SARS‐CoV‐2 carriers were 24, 57 and 64 years old. Five patients had hypertension and were on anti‐hypertensive agents.

Ten patients and two asymptomatic carriers showed undetectable HIV viral load (HIV‐VL) and CD4^+^ T lymphocyte count (CD4 count) between 141 to 817/µL prior to SARS‐CoV‐2 co‐infection. Two HIV‐VL unsuppressed individuals included patient (P5) who presented with a CD4 count of 34/µL without having ever received antiretroviral therapy (ART), and an asymptomatic carrier (P12) who presented with a CD4 count of 29/µL after two weeks of ART. Four co‐morbidity cases presented with a CD4 count less than 200/µL, including two severe cases and two mild cases (All recovered at the end of the study). Although P12 presented with a low CD4 count, he did not even show any symptoms of COVID‐19 or any abnormality in the radiograph. This may indicate that low CD4 count did not inevitably lead to death under HIV and SARS‐CoV‐2 co‐infection. On the contrary, the two deaths occurred in cases who had a relatively high CD4 count (350/µL and 695/µL). This finding is consistent with a previous study, which reported one AIDS/COVID‐19 patient who presented a low CD4 count (13/µL) and recovered finally [2]. Considering that SARS‐CoV‐2 might cause multi‐organ injury through the inflammatory cytokine storm [4], we hypothesized that previous low CD4 count might act a protective role in preventing hyperimmune response. However, our sample size limits the deduction for a generalized conclusion, and hence large‐scale investigation is needed to validate this hypothesis.

The proportion of severe cases (5/11, 45.5%) was higher among the case series than reported in the general population [5]. The COVID‐19 mortality rate (18.2%) was also higher than the overall COVID‐19 mortality (7.7%, 3869/50333) in Wuhan [6]. The median age among the co‐infected cases was older than that of overall patients, and the higher proportion of comorbidities in our patients (8/11, 72.7%) coupled with the senior age might have contributed partially to the higher proportion of severe case and mortality. Living with HIV/AIDS also played a role in leading to comorbidities prior to admission, such as lymphoma and opportunistic infection (pneumocystis pneumonia, PCP).

Reports from laboratory examinations at admission showed leukopenia in six cases and lymphopenia in discrepant six cases. Although the exact immune‐pathogenesis of the decrease in lymphocytes in peripheral blood remains unclear, lymphopenia is also reported as a frequent presentation in HIV negative COVID‐19 patients, especially in severe cases [5, 7]. The pattern of immune cells in AIDS/COVID‐19 patients showed minor differences from that of overall COVID‐19 patients [8]. However, patients’ CD4 count, HIV‐VL and inflammatory cytokines were not tested on the admission, which prevented us from knowing more about the immune status at the early stage of SARS‐CoV‐2 infection in PLHWA. Another limitation should be mentioned that only one ART naïve COVID‐19 case was enrolled in our investigation, although knowing the COVID‐19 prognosis in ART naïve patients is critical. However, together with the case series published earlier [3], the previous immune status and the clinical course of all the patients in this study still help to sum up some experiences in treating COVID‐19 in PLWHA.

The COVID‐19 pandemic has had a significant impact on people around the world, including PLWHA, and continues to grow. Health facilities should endeavour to maintain undisputed antiretroviral supply to PLWHA during the pandemic phase to ensure retention in care and treatment success. Co‐infected patients who presented with low CD4 count and high HIV‐VL, as well as opportunistic infections, should be considered for active treatments as they still have a chance to recover by proper treatment.

## COMPETING INTEREST

None declared.

## AUTHORS’ CONTRIBUTIONS

WT and KL both have full access to all of the data. WG, YF, WT and KL conceived the research. FM, QZ, LL, PM and MG collected all the data. WG, YF, and KL analysed the data and drafted the manuscript. All authors have read and approved the final manuscript.

## References

[1] Jiang H , Zhou Y , Tang W . Maintaining HIV care during the COVID‐19 pandemic. Lancet HIV. 2020;7(5):e308–9. 10.1016/S2352-3018(20)30105-3 PMC7239666 32272084

[2] Blanco JL , Ambrosioni J , Garcia F , et al. COVID‐19 in patients with HIV: clinical case series. Lancet HIV. 2020;7(5):e314–6. 10.1016/S2352-3018(20)30111-9 PMC7159872 32304642

[3] Zhu F , Cao Y , Xu S , Zhou M . Co‐infection of SARS‐CoV‐2 and HIV in a patient in Wuhan city, China. J Med Virol. 2020;92(6):529–30. 32160316 10.1002/jmv.25732 PMC7228399

[4] Cao X . COVID‐19: immunopathology and its implications for therapy. Nat Rev Immunol. 2020;20(5):269–70. 32273594 10.1038/s41577-020-0308-3 PMC7143200

[5] Wang D , Hu B , Hu C , Zhu F , Liu X , Zhang J , et al. Clinical characteristics of 138 hospitalized patients with 2019 novel coronavirus‐infected pneumonia in Wuhan, China. JAMA. 2020;323(11):1061–69. 10.1001/jama.2020.1585 PMC7042881 32031570

[6] Xinhua . Chinese mainland reports 82,692 overall confirmed COVID‐19 cases as Wuhan revises figures: official. Published 2020 Apr 17 [cited 2020 Apr 19]. Available from: http://www.xinhuanet.com/english/2020‐04/17/c_138985339.htm

[7] Guan W‐J , Ni Z‐Y , Hu Y , Liang W‐H , Ou C‐Q , He J‐X , et al. Clinical characteristics of coronavirus disease 2019 in China. N Engl J Med. 2020;382(18):1708–20. 32109013 10.1056/NEJMoa2002032 PMC7092819

[8] Wang F , Nie J , Wang H , Zhao Q , Xiong Y , Deng L , et al. Characteristics of peripheral lymphocyte subset alteration in COVID‐19 pneumonia. J Infect Dis. 2020;221(11):1762–9. 32227123 10.1093/infdis/jiaa150 PMC7184346

